# Visible–Near-Infrared Spectroscopy can predict Mass Transport of Dissolved Chemicals through Intact Soil

**DOI:** 10.1038/s41598-018-29306-9

**Published:** 2018-07-25

**Authors:** Sheela Katuwal, Maria Knadel, Per Moldrup, Trine Norgaard, Mogens H. Greve, Lis W. de Jonge

**Affiliations:** 1 Department of Agroecology, Aarhus University, Blichers Allé 20, PO Box 50, DK-8830 Tjele, Denmark; 2 Department of Civil Engineering, Aalborg University, Thomas Manns Vej 23, DK-9200 Aalborg, Denmark

## Abstract

The intensification of agricultural production to meet the growing demand for agricultural commodities is increasing the use of chemicals. The ability of soils to transport dissolved chemicals depends on both the soil’s texture and structure. Assessment of the transport of dissolved chemicals (solutes) through soils is performed using breakthrough curves (BTCs) where the application of a solute at one site and its appearance over time at another are recorded. Obtaining BTCs from laboratory studies is extremely expensive and time- and labour-consuming. Visible–near-infrared (vis–NIR) spectroscopy is well recognized for its measurement speed and for its low data acquisition cost and can be used for quantitative estimation of basic soil properties such as clay and organic matter. In this study, for the first time ever, vis–NIR spectroscopy was used to predict dissolved chemical breakthrough curves obtained from tritium transport experiments on a large variety of intact soil columns. Averaged across the field, BTCs were estimated with a high degree of accuracy. So, with vis-NIR spectroscopy, the mass transport of dissolved chemicals can be measured, paving the way for next-generation measurements and monitoring of dissolved chemical transport by spectroscopy.

## Introduction

The use of agrochemicals has since the green revolution been an integral part of agricultural intensification to meet the increasing demand for agricultural commodities^1,2^. The extensive use of agrochemicals in agriculture causes pollution of water resources, which poses serious threats to aquatic ecosystems, human health, and the environment^2,3^. The occurrence of agrochemicals and/or their degradation products above the permissible limits in drinking water wells has forced numerous wells to be shut down and the implementation of strict regulations on the use of agrochemicals in the EU countries^3,4^. Soil is the most important transport pathway for agrochemicals to groundwater. Although soil is acknowledged as fundamental for agricultural production, it is equally important for its ability to filter nutrients and pollutants, and for storing and recycling organic material (or carbon)^5,6^. This ability is dependent on the soil’s properties and their interactions and is influenced by how soils are used and managed^7,8^.

Understanding the leaching of dissolved chemicals (solutes) to groundwater and being able to measure and model it is important for health and the environment. The rate and the amount of dissolved chemicals transported through the soil is largely governed by the arrangement of particles and pore networks within the soil, also referred to as the soil structure/architecture^9^. Soil structure is a very dynamic property since it is influenced by basic soil properties such as texture, organic matter, carbonates and metal oxides, the climate, and the land use and management practices^10^. Depending on the soil structure, at near water-saturated conditions, water and dissolved chemicals can, either be transported evenly through the soil, or rapidly through specific pathways in the soil with various degrees of mass exchange between the soil matrix and the transporting pathways. Various solute transport models have been developed to account for different transport processes and facilitate the prediction of the transport of dissolved chemicals through soils^11,12^. Despite our current understanding of the transport processes of water and dissolved chemicals through soils and the availability of various solute transport models, accurate predictions of solute transport is not yet possible. One of the major challenges is to obtain an accurate range of parameters for use in solute transport models to account for the spatial differences in transport properties. Breakthrough curves (BTCs) obtained from column experiments in the laboratory are usually used for deriving estimates of the parameters for solute transport models. However, performing solute transport experiments with a high spatial resolution to account for the spatial diversity in the transport properties is unfeasible.

**Figure 1:**
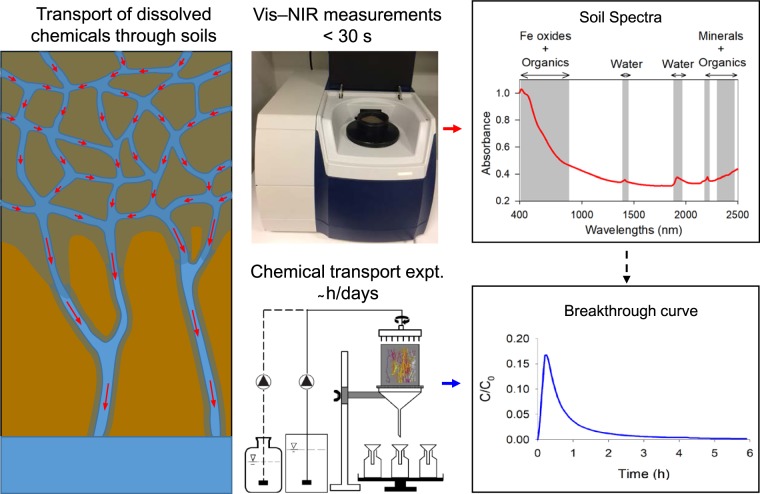
Conceptual representation of the objective of the study. The transport of water and dissolved chemicals through soil is highly complex and is mostly governed by the soil structure/architecture, i.e., the pore-particle network (left figure). The objective of the study was to predict the complex breakthrough curve (lower figures) by the rapid visible–near-infrared (vis-NIR) spectroscopy (upper figures).

In soil science, pedotransfer functions are used to estimate soil properties or model parameters that are difficult to measure, expensive and/or time- and labour-consuming, using readily available soil information^13,14^. There have been only few attempts to develop pedotransfer functions to estimate the parameters for solute transport models using basic soil properties^13,15^. However, obtaining many of the basic soil properties to cover the spatial variability in the landscape is a challenge. In recent years, soil spectroscopy (visible, near- and/or mid-infrared) has proven to be a useful analytical tool for rapid and precise estimation of numerous soil physical, chemical and biological properties such as clay content and mineralogy, soil organic carbon, N, extractable P, K, Fe, soil pH and CEC^16–18^. McBratney *et al*.^19^ suggested the use of soil spectroscopy to bridge the gap between pedotransfer functions and data availability for the prediction of soil functional properties. However, they also reported large uncertainties in the prediction of the desired soil property due to the buildup propagation of uncertainties in the calculation. Recent studies^20,21^ have shown that soil spectroscopy can directly be used to estimate soil hydraulic properties with prediction uncertainties comparable to those obtained using pedotransfer functions. In this study, for the first time ever, we set visible–near-infrared (vis–NIR) spectroscopy the challenge of providing fast estimates of mass transport of dissolved chemicals through intact soils (Fig. 1).

## Dissolved Chemical Mass Transport Through Soils

Flow and transport processes are often studied in the laboratory on soil columns using BTCs. BTCs are obtained from solute transport experiments where the concentrations recovery of the solute applied to one end of the column are measured at the other end at different time intervals. BTCs with early breakthrough, sharp peaks and significant tailing are related to preferential flow^22^ which is the rapid transport of water and solutes through a few preferential pathways while bypassing most of the soil matrix^23^. Preferential flow or non-uniform flow is often associated with heterogeneities in the soil caused by e.g., macropores (inter-aggregate pores, biopores, cracks and fissures)^12^. Uniform flow is related to homogeneous soils and is characterized by bell-shaped BTCs with late breakthrough of solutes.

**Figure 2:**
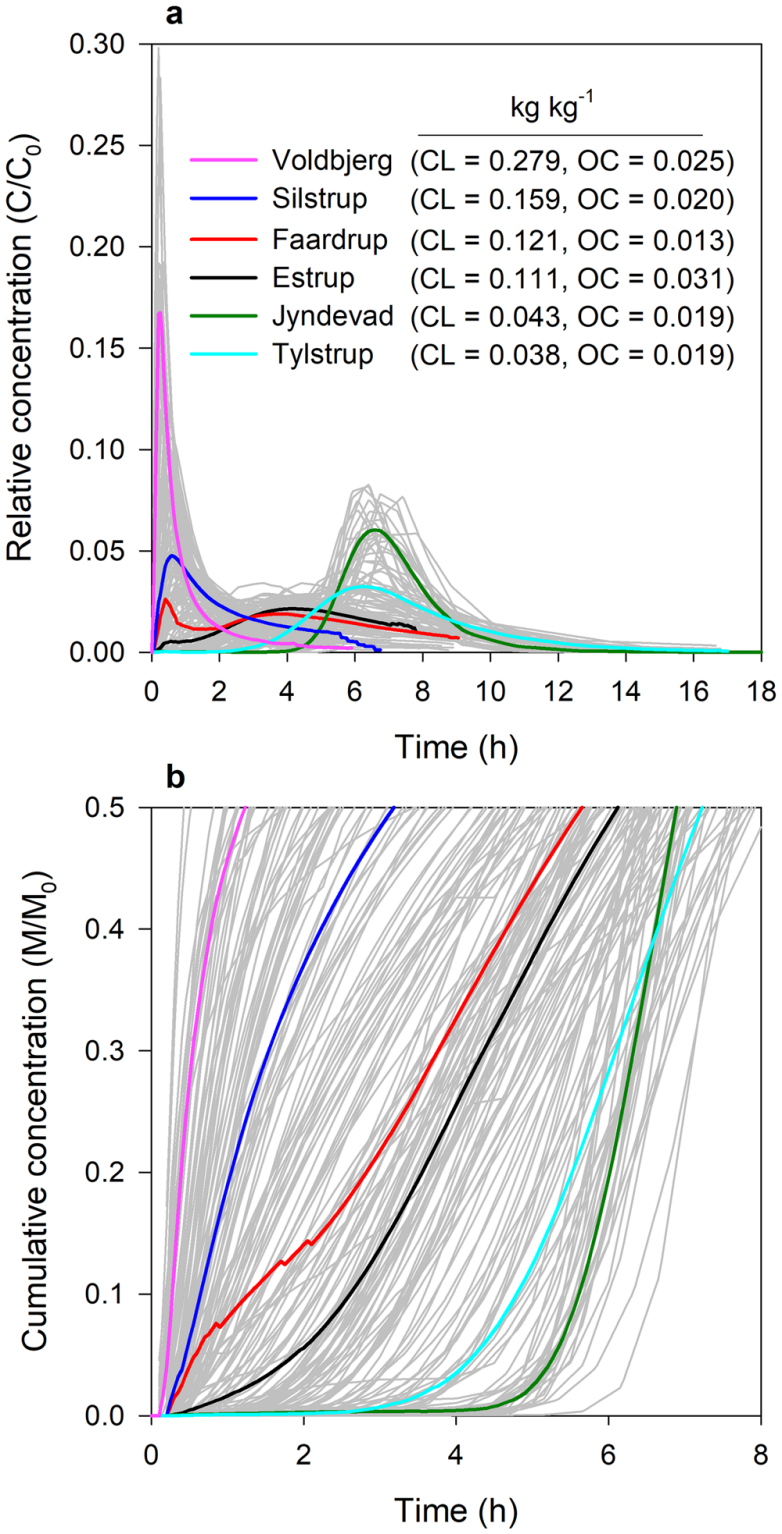
Field-average breakthrough curves. The field-average breakthrough curves along with the 181 individual column breakthrough curves in grey colour in the background expressed as: (**a**) the relative concentration versus time and (**b**) the cumulative mass concentration versus time. CL and OC represent the soil clay content (fraction of soil minerals <2 µm) and soil organic carbon content in kg kg^−1^.

Figure 2 shows the field-average BTCs expressed as the relative concentration (Fig. 2a) or the cumulative concentration (Fig. 2b) of the tracer (tritium) recovered during the column experiments for six fields in Denmark with varying texture and organic carbon (OC) contents (Supplementary Fig. 1). Significant differences in the shapes of the BTCs ranging from strong preferential flow in Voldbjerg clayey soils to uniform/homogeneous flow in Jyndevad sandy soils were observed. Large variations in the shapes of BTCs within the fields were also present (Supplementary Fig. 3). The degree of preferential transport generally decreased with decreasing clay content. However, for the Estrup field with a gradient in OC (0.018–0.084 kg kg^−1^) and a clay content similar to Faardrup, the BTCs were characterized by a lower degree of preferential transport than the Faardrup soils.

Soil texture (especially the type and amount of clay) and OC are important for soil aggregation and structure^10^. Soils with high clay content can contain cracks and fissures due to wetting-drying and freeze-thaw cycles which can result in heterogeneity of soil structure and thus preferential transport^24^. Besides clay content^25,26^, organic carbon content^27^, water saturation^28^ and bulk density^29^ also influence the degree of preferential transport through soils. However, many of these soil properties such as organic matter and bulk density^27,30^, bulk density and saturation ratio^30^ are correlated. Based on X-ray-computed tomography analysis of soils, Katuwal *et al*.^31^ reported that the strength of preferential, and especially the initiation of macropore flow, is influenced by the conductivity or the porosity of the soil matrix which also determines the degree of saturation^30^. Naveed *et al*.^32^ measured soil-moisture retention curves for soils with a clay gradient between 11 and 46% and observed that for soils with 11% clay the pore networks were dominated by pores >3 µm, which were also characterized by a broad range of pore sizes. With increasing clay content the distribution of pores >3 µm narrowed and the overall pore networks were increasingly dominated by pores <3 µm, resulting in diminishing rates of air conductivity with increasing clay content. The presence of macropores in soils with a high clay content (i.e., less conductive soil matrix) thus directs water and solutes towards the macropores, resulting in preferential flow. Conversely, a lack of macropores in such soils can cause ponding at high irrigation rates and such soil columns are usually excluded from further analysis^28,33^. Organic carbon, on the other hand, promotes soil aggregation and increases homogeneity in the soil structure, thus reducing preferential transport through soils^27^, which was also represented by the BTCs of the Estrup soil.

## Variation in Soil Spectra

The spectral data obtained from a soil sample is the result of the interaction of the incident radiation with the molecules/molecular bonds present in the soil. Different molecules present in soil absorb energy at specific wavelengths in the visible and the near-infrared range. The absorption of energy in the visible range is primarily due to electronic transitions of atoms whereas in the near-infrared range it is due to overtones and combinations of fundamental vibrations in the mid-infrared range associated with molecular bonds such as (CH, OH, NH, SO_4_, and CO_3_ groups)^17,34^. The spectral data, thus contain information that reflect the soil’s composition and its properties, which with the use of chemometrics can be used for the simultaneous estimation of several properties such as texture, clay mineralogy, organic and inorganic carbon, CEC, pH, moisture content, mineral oxides and others with a high degree of accuracy^16,17^.

**Figure 3:**
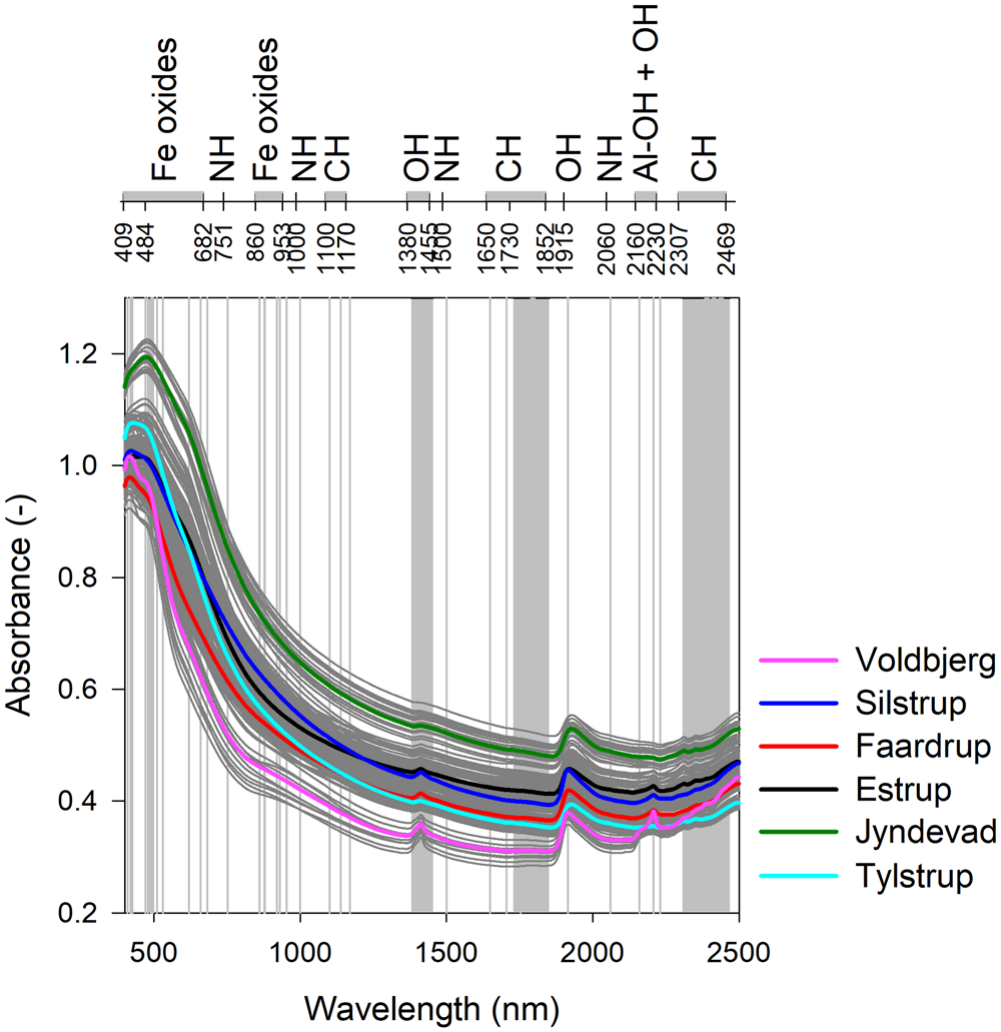
Field-average visible–near infrared spectra. The field-average visible–near infrared spectra along with the spectra for the 181 soils in grey in the background. The vertical lines and bars denote specific absorption bands for the different bonds present in soil, which is specified in the top x-axis.

Figure 3 shows the average field spectra for the studied soils along with the common bonds/functional groups present in soil and their corresponding wavelengths in the visible–near-infrared region^35^. Variations in the overall absorbance and the intensity of absorbance at specific wavelengths or bands were observed both among the fields and within the fields. The coarse-textured Jyndevad soils had higher absorbance related to the scattering effect associated with coarse fractions in soil and/or a higher degree of coating with organic matter^18^. The fine-textured Voldbjerg soil had lower absorbance associated with the finer texture and the lighter colour. In general, the soils from different fields showed variations in the absorbance at wavelengths associated with iron oxides (400–500 nm), clay minerals (OH bond: 1400 nm, 1900 nm, Al-OH bond: 2200 nm and organic matter (CH bond: 2300–2400 nm) present in the soil^36,37^. Variations in the spectra within the fields were related to the variations in the soil properties (texture and OC) within the fields (Supplementary Fig. 5). Large within-field variations in the spectra were obtained for Voldbjerg soils with its large variation in clay content and Estrup soils with its variation in organic carbon content.

## Prediction Of Arrival Times And Field-Average Btcs

An arrival time represents the time it takes for a definite fraction of solute to be recovered from the end of a soil column and is obtained from the cumulative BTCs (Fig. 2b). Arrival times from 5–50% were obtained from the BTCs and estimated using vis–NIR spectroscopy data by performing partial least squares (PLS) regression with10-fold cross-validation. No attempts were made to fit solute transport models to the BTCs and estimate the fitted parameters since the dominant flow process in the soils varied widely from strong preferential to clear uniform flow, suggesting that a single model could not be used for fitting the BTCs^11^.

**Figure 4:**
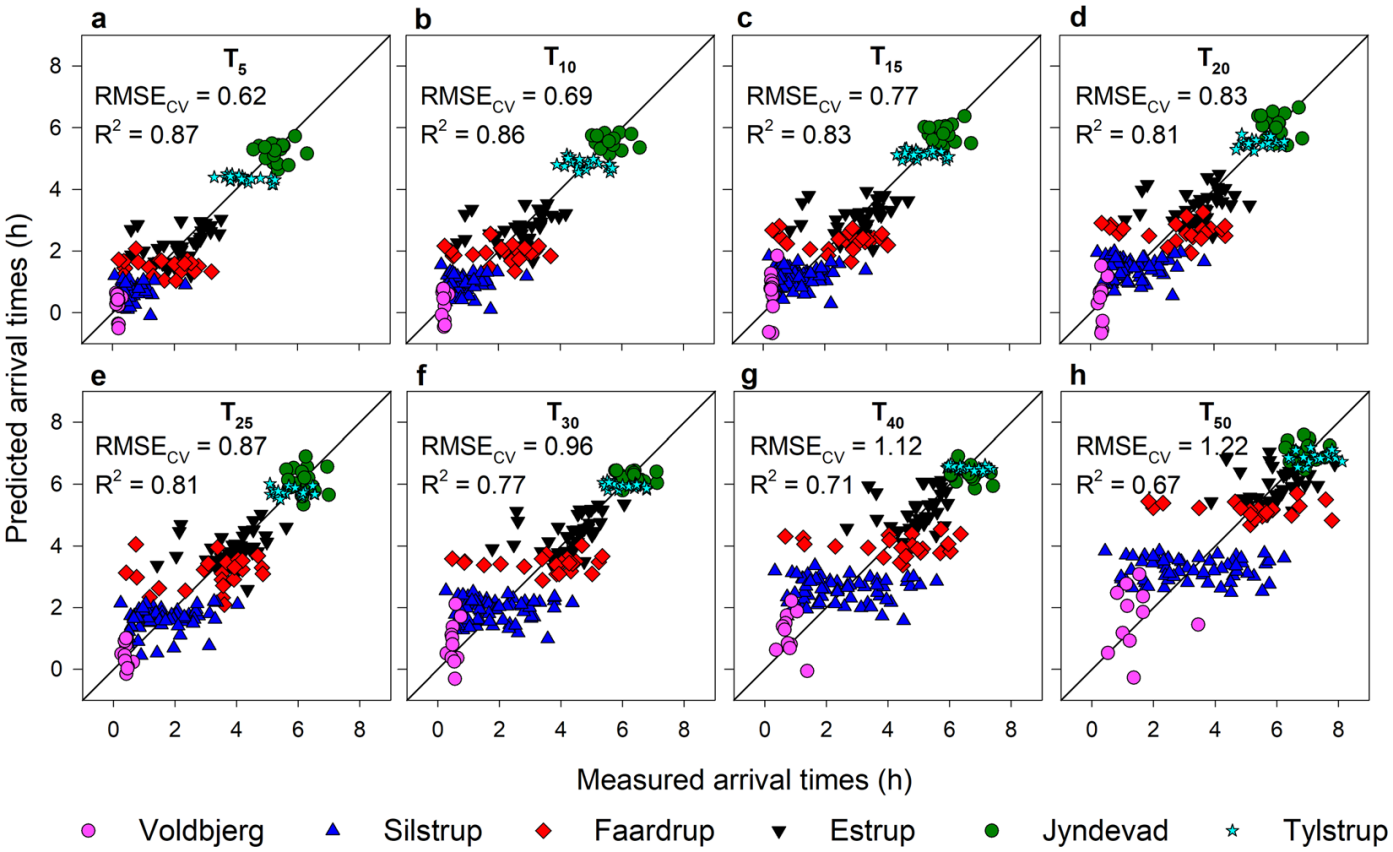
Predicted versus measured arrival times. (a–h) Performance of 10-fold cross-validation for the prediction of various arrival times (T_5_–T_50_) using vis–NIR spectroscopy and partial least squares regression against arrival times measured in the laboratory. RMSE_CV_ denotes the root mean square error of cross-validation and R^2^ denotes the coefficient of determination.

**Figure 5:**
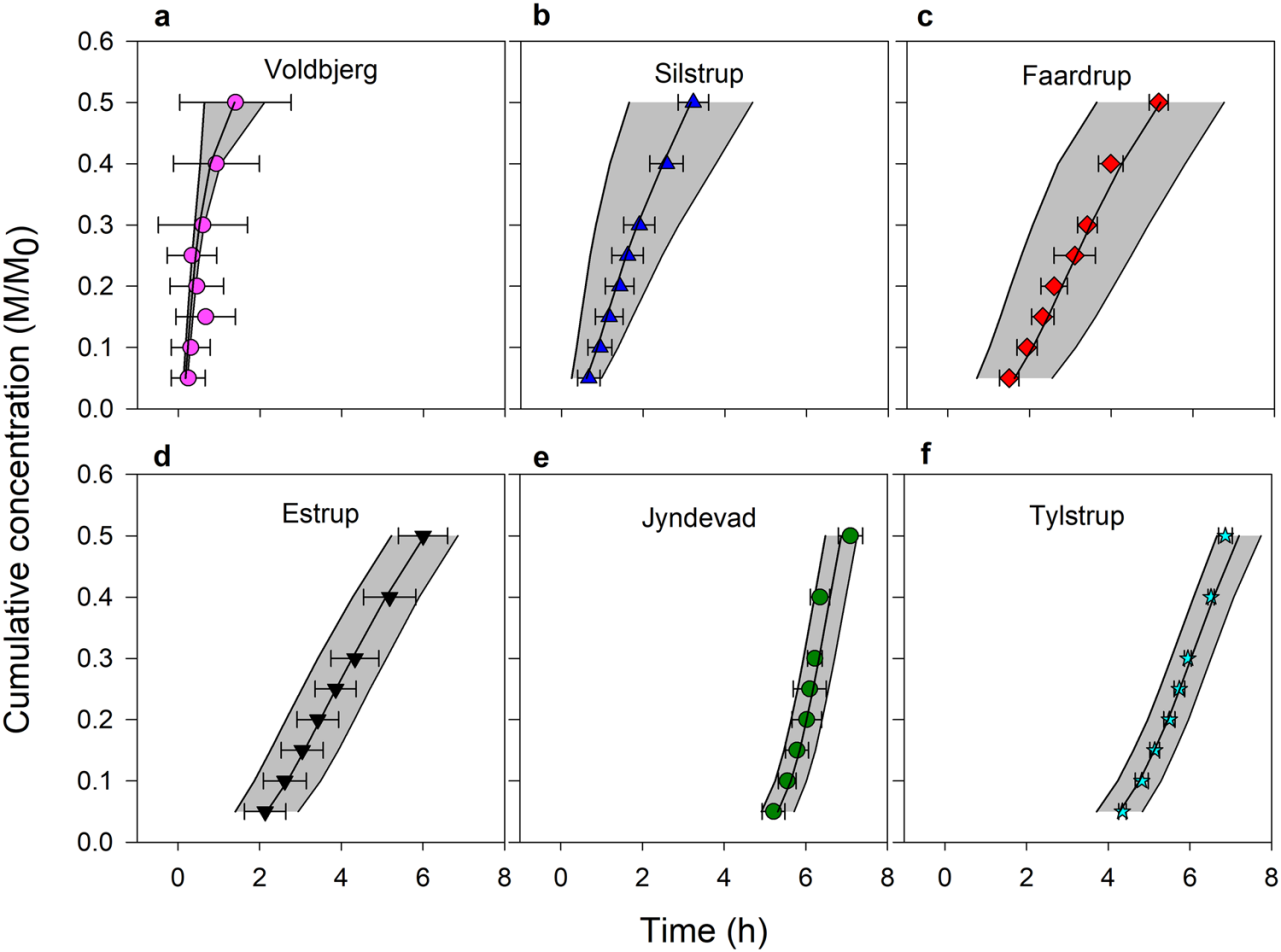
Predicted versus measured breakthrough curves. (a–f) Comparison of predicted BTCs using vis–NIR spectroscopy with measured BTCs in the laboratory for various fields. The symbols and the error bars represent respectively the average values and one standard deviation for the predicted BTCs. The black line and grey background represent respectively the average and one standard deviation of the measured BTCs.

Figure 4(a–h) shows the prediction results for various arrival times of the tracer (5, 10, 15, 20, 25, 30 and 50% and denoted by T_5–50_ respectively) using vis–NIR spectra. A comparison of the estimated field-average BTCs to the measured field-average BTCs for the six fields is presented in Fig. 5. The estimation accuracies were higher for the early arrival times as compared to the later arrival times (Table 1). Scatter along the 1:1 line was observed for soils from all the fields and increased with the later arrival times. Strong agreements between the measured field-average BTCs and estimated BTCs using vis–NIR spectroscopy were obtained for the different fields (Fig. 5a–f). There was a greater variation in the predicted arrival times compared to the measured values for the Voldbjerg soils (Figs 4 and 5a). Despite the large variation in clay content, the Voldbjerg soils had less variation in BTCs, basically due to the dominance of preferential flow through macropores for the experimental conditions. For soils from the other fields, especially Silstrup, Faardrup, and Tylstrup, vis–NIR spectroscopy did not result in the large variances in the arrival times as with the measured values (Figs 4 and 5). Such underestimation of variances in the estimated soil water retention using different pedotransfer functions has also been reported by Pringle *et al*.^38^. The Silstrup, Faardrup and Tylstrup fields are characterized by small gradients in clay and OC content (Silstrup: 0.147–0.197 kg kg^−1^ clay and 0.017–0.022 kg kg^−1^ OC, Faardrup: 0.101–0.192 kg kg^−1^ clay and 0.010–0.015 kg kg^−1^ OC and Tylstrup: 0.034–0.045 kg kg^−1^ clay and 0.017–0.020 kg kg^−1^ OC). Previous studies by Soares *et al*.^39^ and Norgaard *et al*.^40^ found that the degree of preferential transport in the Faardrup and Silstrup fields correlated best with bulk density. Bulk density in the soils from the two fields was to some extent correlated to OC. However, soil bulk density is also influenced by other factors such as agricultural management practices and biological activities, which cannot be detected by vis–NIR spectroscopy.

**Table 1:** Results of cross-validation using visible–near-infrared spectroscopy partial-least-squares regression (PLSR) models for prediction of various arrival times dataset.

Arrival times	Preprocessing	Factors	Cross-validation (N = 181)			
			RMSE_CV_	R^2^	RPIQ	Bias
T_5_ (h)	2^nd^ derivative (17,9)	7	0.62	0.87	4.1	0.00
T_10_ (h)	2^nd^ derivative (7,17)	7	0.69	0.86	4.1	−0.01
T_15_ (h)	2^nd^ derivative (5,25)	7	0.77	0.83	4.0	0.01
T_20_ (h)	2^nd^ derivative (19,5)	6	0.83	0.81	3.9	0.00
T_25_ (h)	2^nd^ derivative (25,31)	10	0.87	0.81	4.0	−0.01
T_30_ (h)	2^nd^ derivative (9,19)	5	0.96	0.77	3.8	0.01
T_40_ (h)	2^nd^ derivative (7,27)	4	1.12	0.71	3.4	0.00
T_50_ (h)	2^nd^ derivative (5,19)	6	1.22	0.67	3.1	0.00

RMSE_CV_ denotes the root mean square error of cross-validation, R^2^ is the coefficient of determination, RPIQ denotes the ratio of performance to interquartile range and N is the number of samples.

^Ŧ^2^nd^ derivative (w, s) refers to preprocessing with second derivative of the spectra using window/gap size of w, i.e., the number of data points across which the derivative is taken and segment, s, is the number of data points/segments across which smoothing/averaging is performed prior to derivative.

For the same 181 soils, Karup *et al*.^33^ showed that the range of arrival times (5–50%) of the applied solute can be predicted using basic soil properties such as the volumetric content of fines (clay and silt), which is a ratio of the gravimetric mass fraction of fines times the soil bulk density to the particle density of fines. However, this requires that quantitative information of the fines content and bulk density of the soils is available for such estimations, which is resource-demanding. The RMSE values for the arrival times obtained in this study using vis–NIR spectroscopy are similar to those obtained by Karup *et al*.^33^. However, it should be noted that the results in this study are based on a 10-fold cross-validation, whereas the RMSE values in Karup *et al*. are based on regression analyses on the entire data set without any validation. The cross-validation results (also the calibration-validation results in the supplementary section, Supplementary Figs 7 and 8, and Supplementary Table 2) of this study show that vis–NIR could be used for rapid and acceptable predictions of the arrival times. To extend vis–NIR calibration models to other fields, does however require additional measurements from the target fields before the models can be utilized successfully. Good prediction performance of the arrival times also means that vis–NIR spectroscopy can function as a rapid alternative for estimating mass solute BTCs for various direct and indirect solute transport applications such as optimization of parameters in solute transport modelling, and risk assessment of pollutants. Even though there is an issue with the underestimation of the within-field variances with vis–NIR spectroscopy, the efficiency of this technology in terms of cost and speed of measurement may outweigh a few expensive and precise measurements of soil properties which usually have large spatial variability^41^. Nonetheless, approaches for reducing the estimation error resulting from differences in soil structure that cannot be captured by vis–NIR spectroscopy should be further investigated. Integration of vis–NIR spectroscopy with other readily available information such as soil structure information based on soil surveys or quick field tests may improve the prediction accuracies and should be explored. Moreover, as the user community within the area of soil science is increasing, specific standard procedures for measurements and the specification of equipment used should be followed in order to merge data from different sources^42^. A few-seconds’ spectroscopic reading combined with PLS analyses can likely provide rapid, inexpensive predictions of not only static soil properties but also dynamic soil properties like chemical mass transport as a platform for next-generation soil measurements and monitoring.

## Methods

### Field sites and soils

The soils used in the study have already been described in earlier studies^30,31,33,40,43,44^. In this study, 181 soil samples collected from the top 20-cm depth from six different fields in Denmark (Supplementary Fig. 1) were studied. The soils from the different fields varied in texture and organic carbon (OC) content to different degrees. The Voldbjerg field (56° 18′ 71″ N, 8° 54′ 87″ E, 1.4 ha) is characterized by a gradient in clay content (0.196–0.426 kg kg^−1^) where the texture of the topsoil varied from loam to silty clay while OC varied only between 0.017–0.020 kg kg^−1^ (ref.^33^). The topsoil in the Silstrup field (56° 55′ 56″ N, 8° 38′ 44″ E, 1.7 ha) is a sandy clay loam/sandy loam with 0.147–0.197 kg kg^−1^ clay and 0.017–0.022 kg kg^−1^ OC^40^. The topsoil in the Faardrup field (55° 19′ 1″ N, 11° 20′ 34″ E, 2.3 ha) is a sandy loam soil with 0.101–0.192 kg kg^−1^ clay and 0.010–0.015 kg kg^−1^ OC^31^. The Estrup field (55° 29′ 10″ N, 9° 04′ 9″ E, 1.3 ha) is a heterogeneous field with a gradient in OC, where the topsoil is a sandy loam soil with 0.055–0.140 kg kg^−1^ clay and 0.018-0.084 kg kg^−1^ OC^30^. Both Jyndevad (54° 53′ 37″ N, 9° 07′ 12″ E, 2.4 ha) and Tylstrup (57° 10′ 47″ N, 9° 57′ 24″ E, 1.1 ha) have coarser textures and were more homogeneous than the other fields. The topsoil in Jyndevad is a sandy soil with a clay content of 0.037–0.052 kg kg^−1^ and OC of 0.014–0.025 kg kg^−1^ (ref.^44^). The topsoil in Tylstrup is a loamy sand with a clay content of 0.034–0.045 kg kg^−1^ and OC of 0.017–0.020 kg kg^−1^ (ref.^33^). The latest agricultural management practices in the fields before sampling included ploughing to 20–25 cm depth and cropping with winter wheat/winter rye and/or spring barley. The soil samples from these fields were collected between 2010 and 2014. At the time of soil sampling the fields had not been ploughed for at least a year.

For the solute transport experiments, intact soil columns (20 cm in diameter and 18–20 cm in height) were collected from the topsoil. Aluminum or steel cylindrical rings (20 × 20 cm) were pushed into the soil using the hydraulic press of a tractor until the soil surface was close to the cylinder rim. The surrounding soil was removed manually to extract the soil columns, which were then trimmed at the bottom, sealed with plastic caps and stored at 2 °C until the experiments were performed.

Bulk soil samples from the same depth (0–20 cm) and sampling points were collected and mixed thoroughly. The soils were air-dried, ground and sieved through a 2-mm sieve. A combination of wet sieving and hydrometer methods following Gee and Or^45^ were used for determining soil texture. The OC content was measured on ball-milled samples with a FLASH 2000 NC organic elemental analyser (Thermo Fisher Scientific Inc., Waltham, MA, USA).

### Dissolved chemical transport experiments

Supplementary Fig. 2 shows the setup for the solute transport experiment. The soil columns were slowly saturated from the bottom with an artificial soil water solution (0.652 mM NaCl, 0.025 mM KCl, 1.842 mM CaCl_2_ and 0.255 mM MgCl_2_; pH = 6.38; EC = 0.6 mS cm^−1^) and drained to a matric potential of -10 cm at the bottom. The drained columns were placed on a 1-mm stainless steel wire mesh to allow free drainage from the bottom of the columns. Irrigation was applied to the top at an intensity of 10 mm h^−1^ with an artificial rainwater solution (0.012 mM CaCl_2_, 0.015 mM MgCl_2_ and 0.121 mM NaCl; EC = 0.025 mS cm^−1^; pH = 6.5) using a rotating irrigation head. The rotating irrigation head consisted of 44 hypodermic needles placed randomly to ensure homogeneity during irrigation on the soil surface. A funnel connected to the wire mesh assembly at the discharge end of the soil column delivered the effluent to bottles placed on a rotating table below the soil columns. The irrigation was applied for 6–18 h after the breakthrough of irrigation water from the bottom of the columns. When the discharge from the soil column was steady, a conservative tracer consisting of tritium solution (^3^H_2_O) applied as a pulse for 10 minutes replaced the irrigation water. The activity of tritium in the effluent collected at different time intervals was analyzed using a liquid scintillation analyzer (Packard 2250 CA, Downers Grove, IL, USA). The concentrations/fractions of tracer transported to the outflow at various time intervals were determined from the effluent. The breakthrough curves (BTCs) were expressed as the relative concentration of the tracer (Fig. 2a) and the cumulative mass of the tracer (Fig. 2b). To obtain the field-average BTCs, first the total duration of the experiment was discretized to a temporal resolution of 0.05 h (3 min.) and the respective relative tracer concentration/cumulative tracer mass was calculated by linear interpolation between each of the two consecutive measurements. The arithmetic average of the interpolated relative tracer concentration/cumulative tracer mass for each field at respective times provided the field-average BTCs. The breakthrough curves of all the soil for the six fields are presented in Supplementary Fig. 3.

### Visible–near-infrared measurements and analysis

The spectra of the soils were measured using a benchtop spectrometer (NIRS DS2500, FOSS, Hillerød, Denmark) (Supplementary Fig. 4). The equipment measures diffuse reflectance within the spectral range of 400–2500 nm with a resolution of 0.5 nm. The instrument was calibrated by scanning a white reference prior to the measurements of the soil samples. About 50 g of air-dried and sieved ( ≤ 2 mm) soil was mixed properly and placed in a rotating quartz sample cup of 70 mm diameter so that the soil completely covered the base of the cup. As the sample cup rotates, the instrument measures the diffuse reflectance four times at seven different positions. The spectral reflectance for each sample is thus an average of 28 spectral measurements. The complete measurement takes about 30 s. The instrument automatically converts the diffuse reflectance (R) measurements into absorbance (A) measurements using the relation A = log (1/R).

The analysis of the vis–NIR data was performed using the “pls package”^46^ in R software. The arrival times of various fractions of the tracer (5, 10, 15, 20, 25, 30 and 50% denoted by T_5–50_, respectively) obtained from the BTCs were predicted using vis–NIR spectral data and partial least squares regression (PLSR) after spectral-pretreatment. The spectral pretreatment included performing second-derivative of the spectra with a gap size of w = 5–25 and a segment size of s = 2–31 for different arrival times (Supplementary Table 2). The gap size denotes the number of data points across which the derivative is taken and the segment, s, is the number of data points/segments across which smoothing/averaging is performed prior to derivative^47^. The prediction was done using a 10-fold cross-validation on the pretreated spectra after obtaining the optimum number of latent variables. In addition, prediction of the arrival times was also obtained by first dividing the total dataset into a calibration (121 samples) and validation (60 samples) dataset. Then the models developed from the calibration dataset were used for predicting the arrival times for the validation dataset. The results for the calibration-validation are presented in the supplementary section (Supplementary Figs 7 and 8; Supplementary Tables 1 and 2). The root mean square error of cross-validation (RMSE_CV_) between the estimated values and the measured values, the coefficient of determination (R^2^), the ratio of performance (RMSE_CV_) to interquartile range (RPIQ)^48^ and the bias between the measured and the predicted values were calculated to evaluate the prediction performance.

## Electronic supplementary material

Supplementary Information
