# Supplementary material for: Visible–Near-Infrared Spectroscopy can predict Mass Transport of Dissolved Chemicals through Intact Soil
Source: Sci Rep. 2018 Jul 25;8:11188. doi: 10.1038/s41598-018-29306-9 (PMC6060133; doi:10.1038/s41598-018-29306-9)
Supplement: Supplementary file 1 — Supplementary Information [file 41598_2018_29306_MOESM1_ESM.pdf]

Supplementary figures and tables

Visible–Near-Infrared Spectroscopy can predict Mass Transport of Dissolved Chemicals through Intact Soil

Sheela Katuwal[1]*, Maria Knadel[1], Per Moldrup[2], Trine Norgaard[1], Mogens H. Greve[1] and Lis W. de Jonge[1]

[1] Department of Agroecology, Aarhus University, Blichers Allé 20, PO Box 50, DK-8830 Tjele, Denmark

*e-mail: Sheela.Katuwal@agro.au.dk

[2] Department of Civil Engineering, Aalborg University, Thomas Manns Vej 23, DK-9200 Aalborg, Denmark

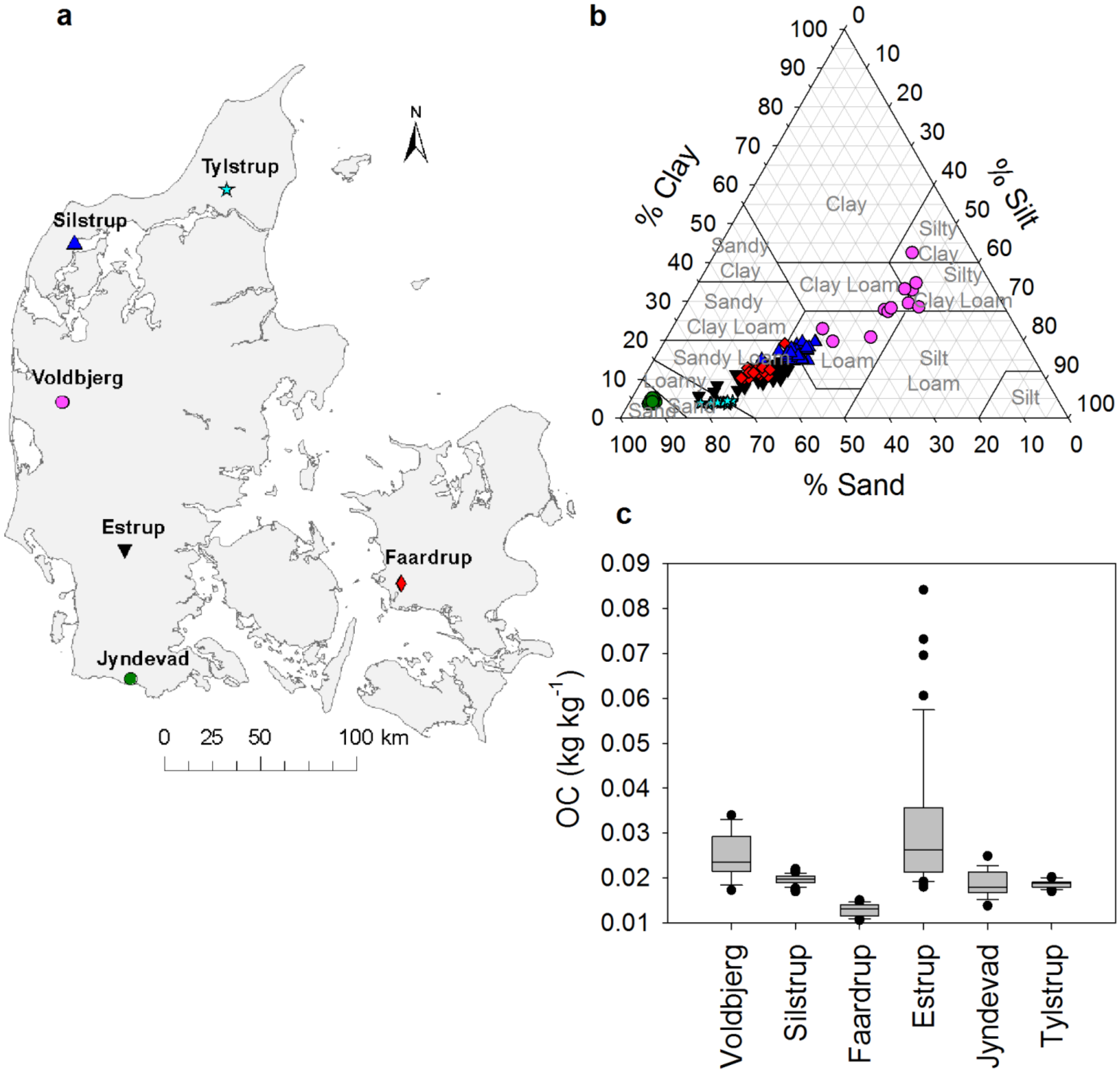

Fig. S1: a) Location of the fields in Denmark. **b)** Variation in soil texture expressed as content (%) of clay (< 2 µm), silt (2–50 µm) and sand (50–2000 µm) in the USDA texture triangle. **c)** Boxplot showing the variation in the OC (kg kg^{-1}) of the soils in the different fields. The bottom and top of the box represent the first and third quartiles of the distribution in OC in each field respectively and the band within the box represents the median value. The bottom and top whiskers represent the 10th and 90th percentile respectively and the point symbols are the values lying outside the 10th and 90th percentile.

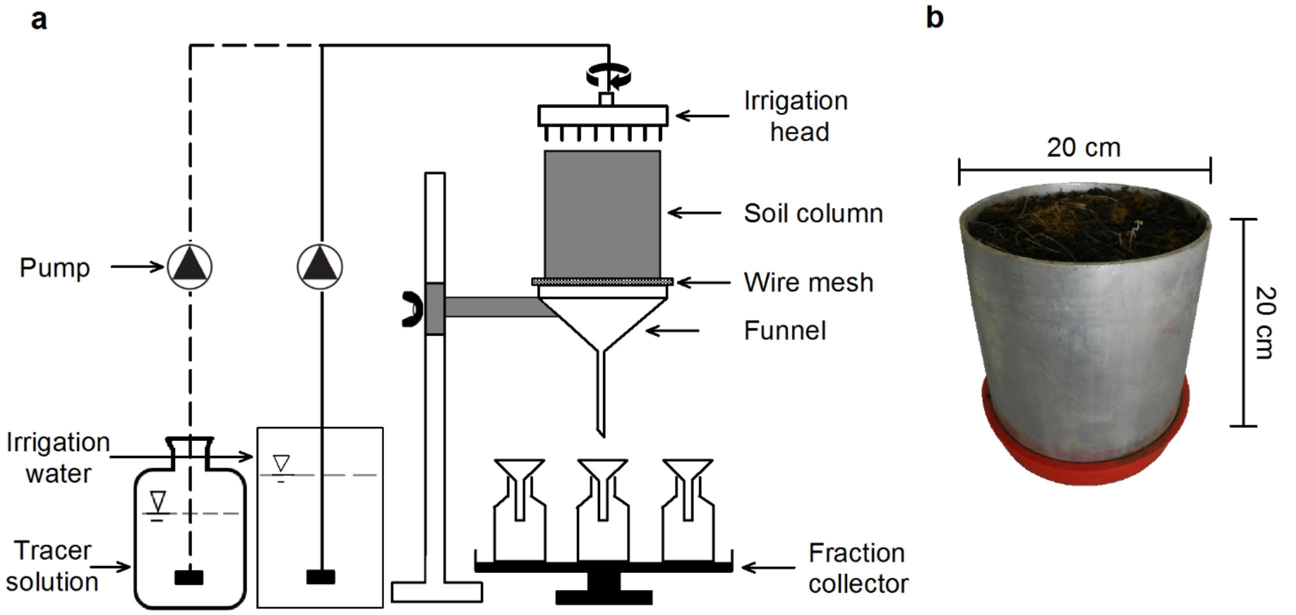

Fig. S2: a) Schematic representation (not in scale) of the experimental setup for the solute transport experiments. The dashed line represents the short duration of the application of the tracer solution during the experiment. **b)** An example of a soil sample (20 cm diameter x 20 cm height) used in the solute transport experiment.

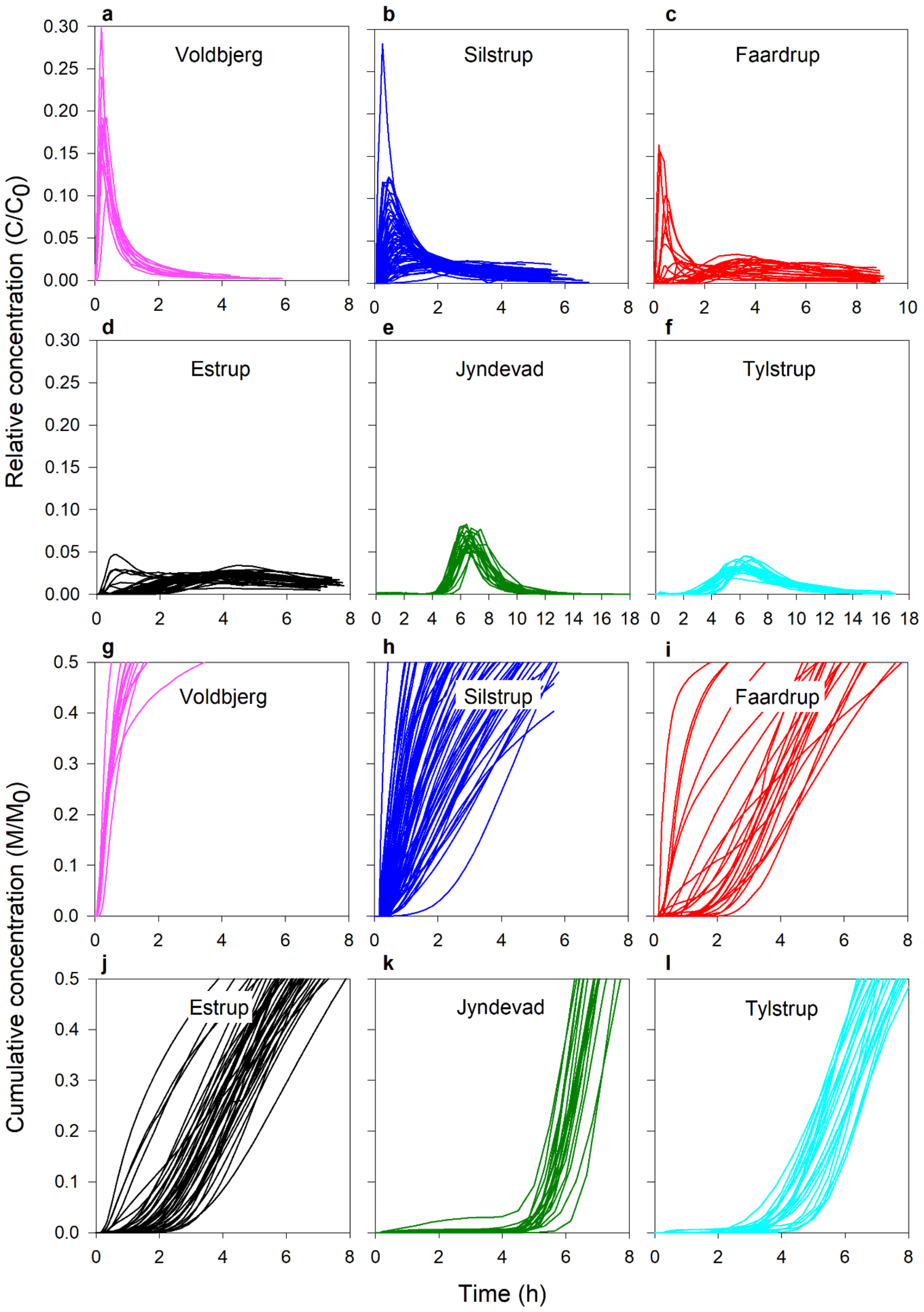

Fig. S3: Breakthrough curves of individual columns for the different fields expressed as: **a–f)** the relative concentration versus time and **g–l)** the cumulative mass concentration versus time.

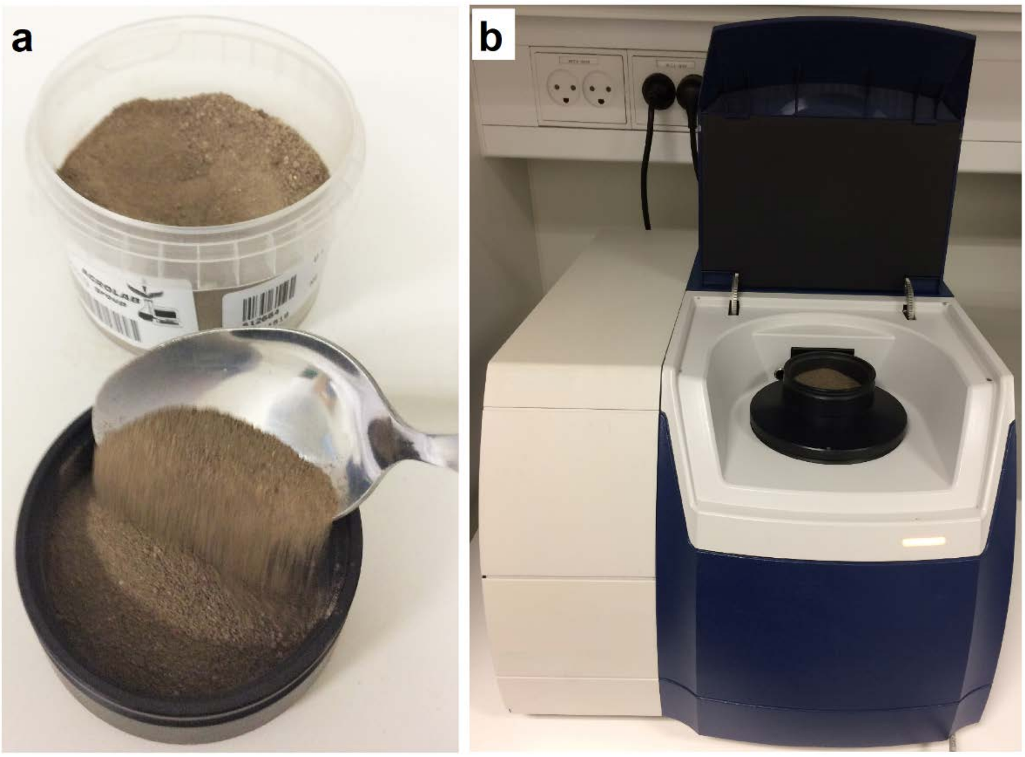

Fig. S4: a) Sample preparation with bulk soil (about 30 g, ≤ 2 mm and air-dried) for scanning using visible–near-infrared spectrometer, and **b)** setup for scanning the prepared soil.

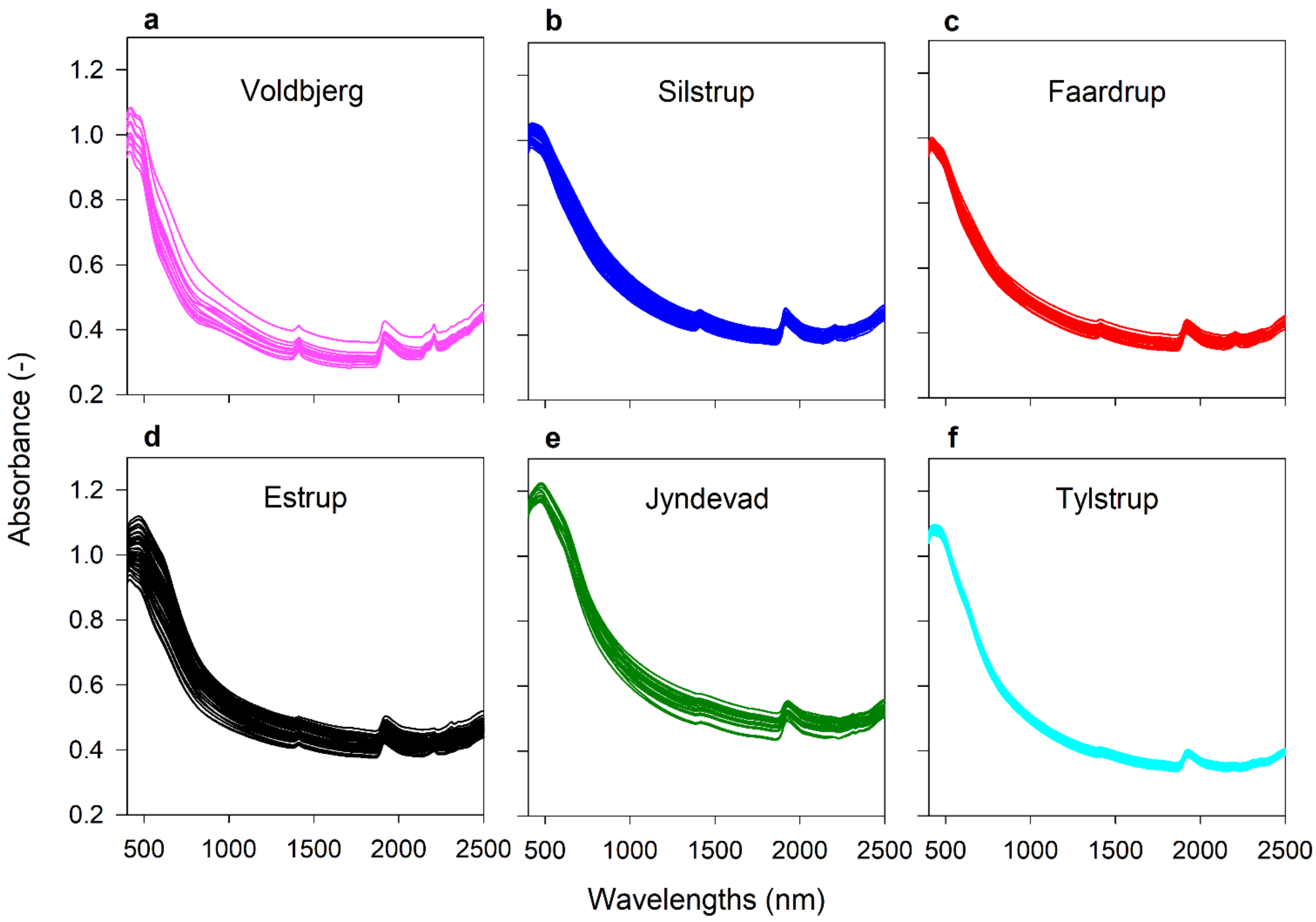

Fig. S5: Visible–near-infrared spectra of the soils in different fields.

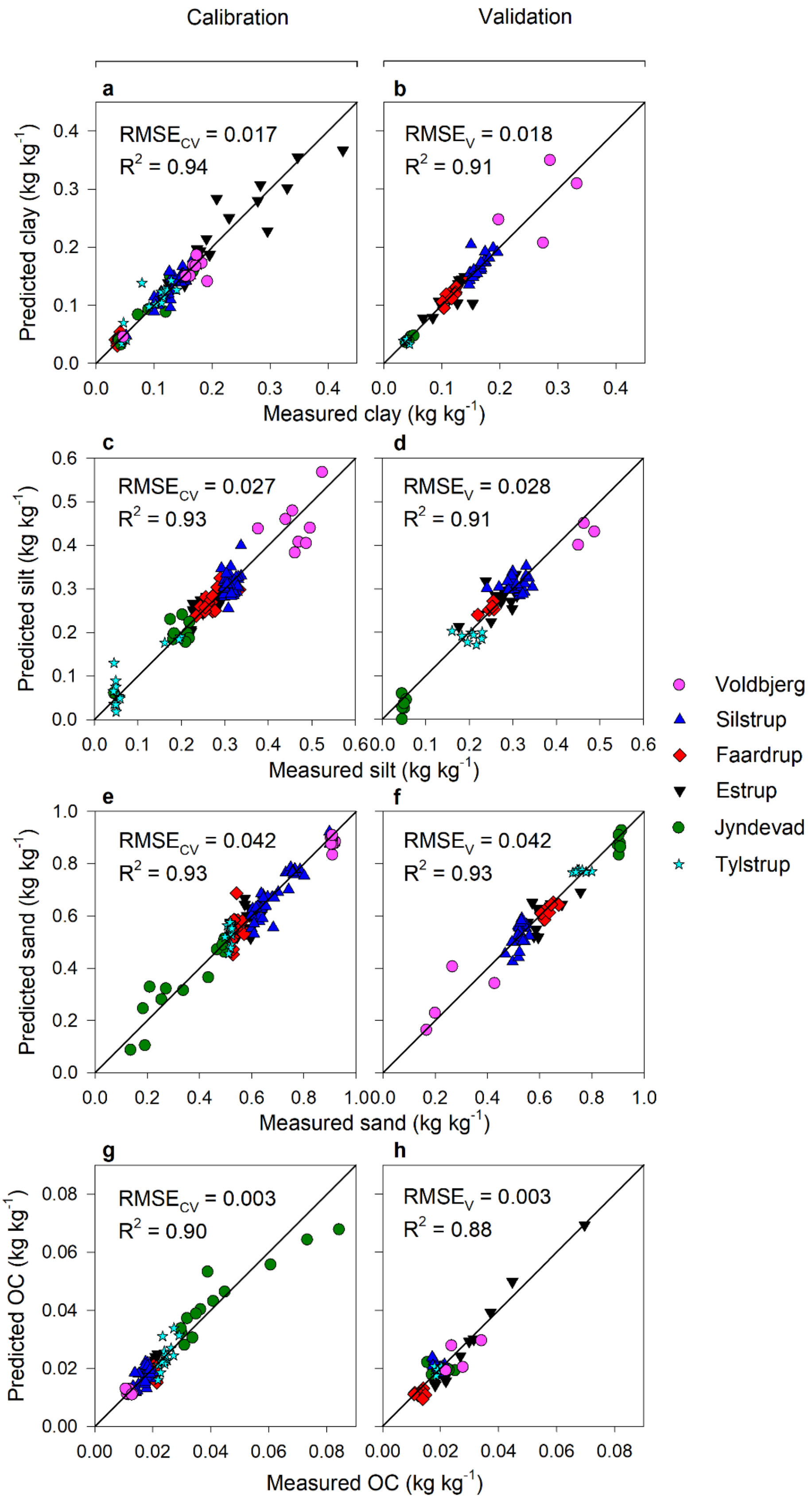

Fig. S6: Predicted versus measured clay, silt, sand and organic carbon (OC) using visible–near-infrared spectroscopy data and partial least squares regression for the calibration dataset (121 soils) and validation dataset (60 soils).

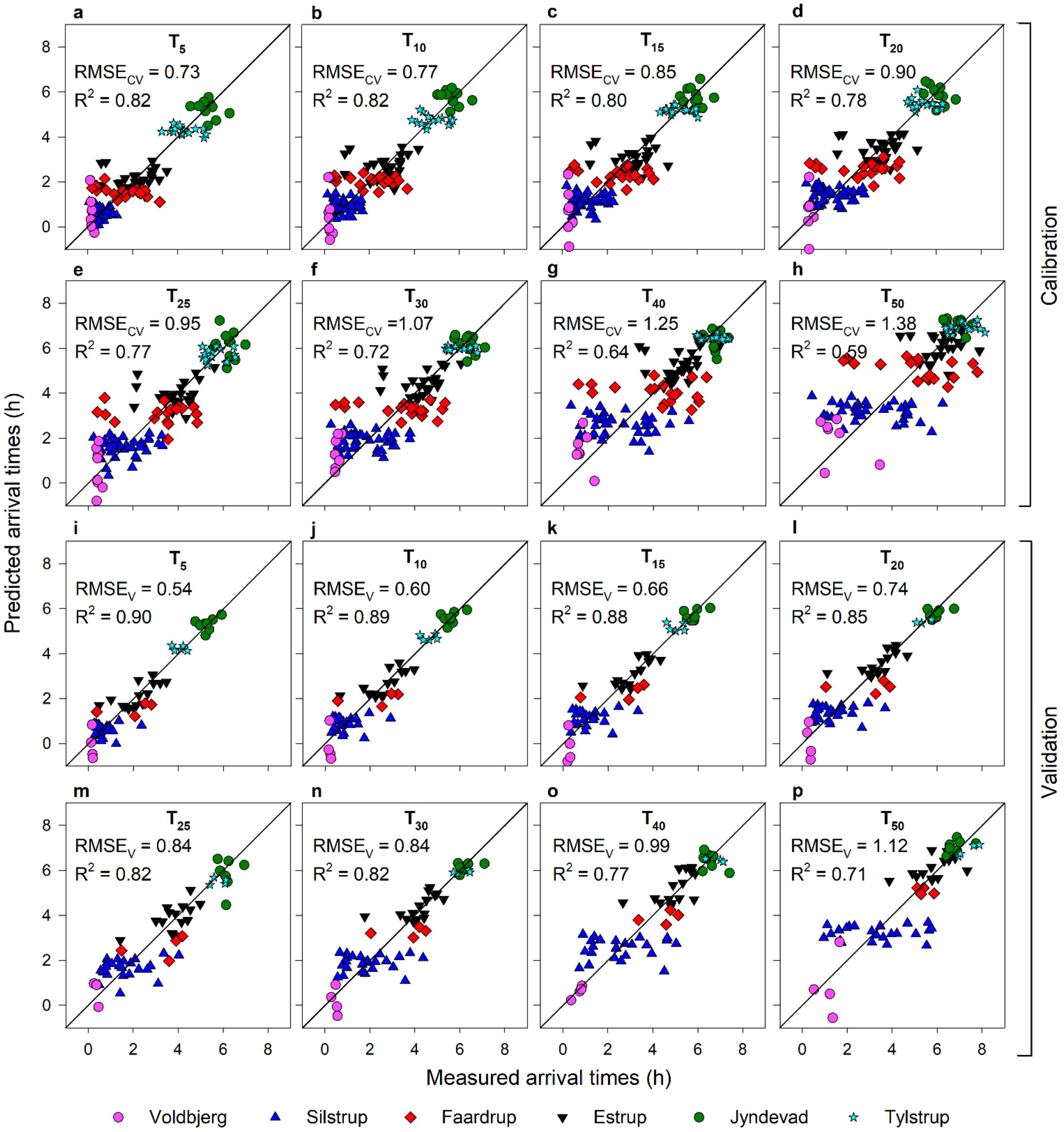

Fig. S7: Prediction of different arrival times (T_5–T_{50}) using visible–near-infrared spectroscopy and partial least squares regression for the calibration dataset (121 soils) and validation dataset (60 soils).

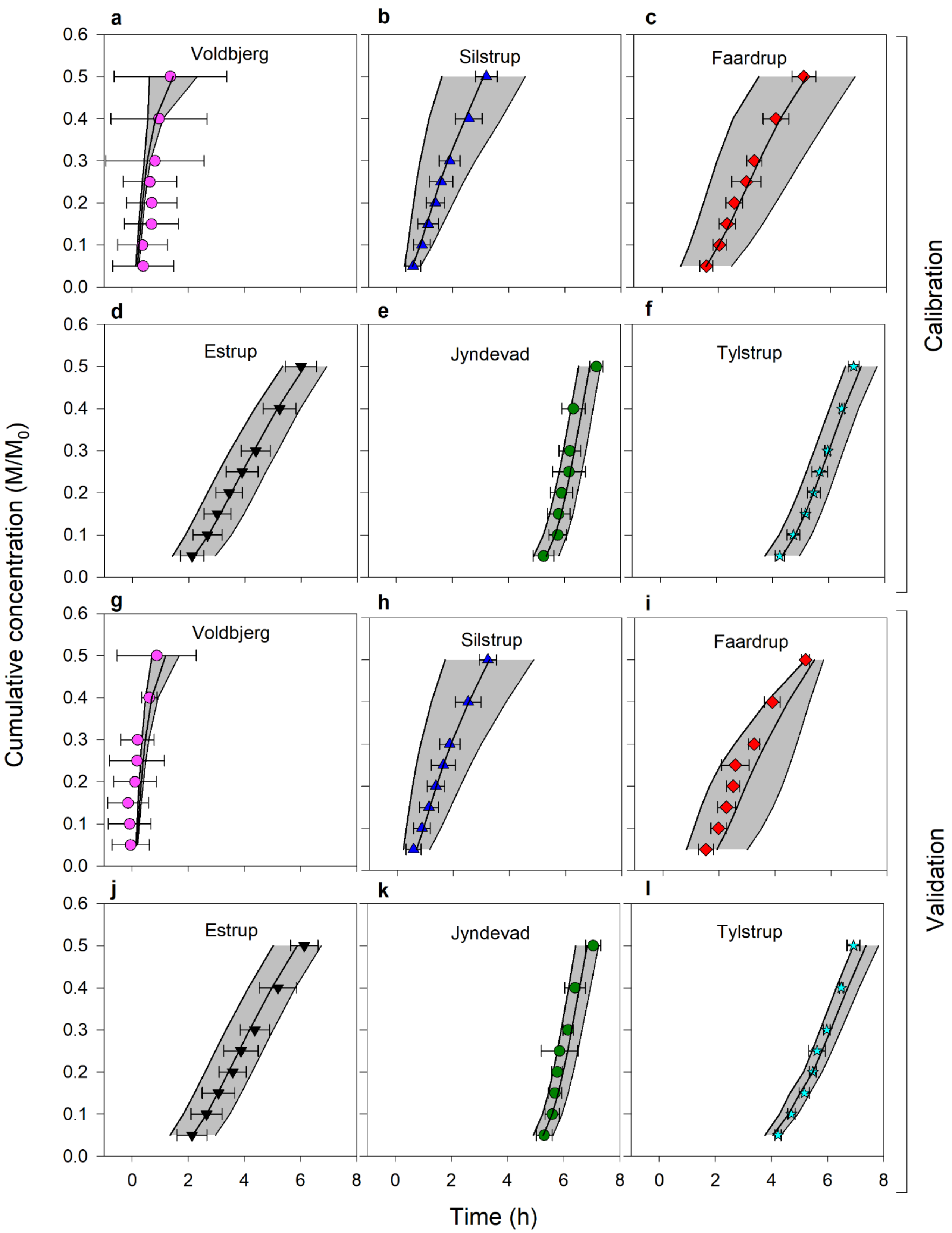

Fig. S8: Predicted versus measured breakthrough curves (BTCs) for the different fields for the calibration dataset (121 soils) and validation dataset (60 soils). The points are the average values and the error bars denote one standard deviation for the predicted BTCs using visible–near-infrared spectroscopy. The black line and grey background respectively represent the average and one standard deviation of the measured BTCs in the laboratory.

Table S1: Statistics of the soil properties (texture and organic carbon (OC)) for the calibration and validation dataset.

Statistics	Calibration dataset (N = 121)				Validation dataset (N = 60)			
	Clay	Silt	Sand	OC	Clay	Silt	Sand	OC
	kg kg^{-1}							
Minimum	0.033	0.043	0.132	0.011	0.036	0.043	0.160	0.011
Q1	0.090	0.220	0.507	0.018	0.080	0.220	0.503	0.019
Q3	0.155	0.298	0.661	0.021	0.158	0.305	0.665	0.022
Maximum	0.413	0.502	0.889	0.084	0.335	0.469	0.899	0.609
Mean	0.124	0.253	0.589	0.021	0.127	0.251	0.579	0.032
σ [Ŧ]	0.064	0.087	0.149	0.010	0.063	0.102	0.168	0.076

[Ŧ]σ denotes the standard deviation

Table S2: Performance of the visible–near-infrared spectroscopy calibration and validation partial-least-squares regression (PLSR) models for prediction of various soil properties for the calibration dataset and validation dataset. $RMSE_{CV}$ denotes the root mean square error of cross-validation for the calibration data, $RMSE_V$ denotes the root means square error of the validation data and N is the number of samples in each dataset.

Soil properties	Preprocessing	Factors	Calibration (N = 121)		Validation (N = 60)			
			$RMSE_{CV}$	R^2	$RMSE_V$	R^2	RPIQ	Bias
**Clay (kg kg^{-1})**	2nd derivative (19,2)Ŧ	4	0.017	0.94	0.018	0.91	4.0	0.001
**Silt (kg kg^{-1})**	2nd derivative (9,13)	7	0.027	0.93	0.028	0.91	3.0	-0.002
**Sand (kg kg^{-1})**	2nd derivative (7,10)	4	0.042	0.93	0.042	0.93	3.6	-0.003
**OC (kg kg^{-1})**	2nd derivative (5,18)	7	0.003	0.90	0.003	0.88	1.2	-0.001
**T_5 (h)**	2nd derivative (17,9)	7	0.73	0.82	0.54	0.90	4.6	-0.07
**T_{10} (h)**	2nd derivative (7,17)	7	0.77	0.82	0.60	0.89	4.6	-0.06
**T_{15} (h)**	2nd derivative (5,25)	7	0.85	0.80	0.66	0.88	4.5	-0.06
**T_{20} (h)**	2nd derivative (19,5)	6	0.90	0.78	0.74	0.85	4.1	0.05
**T_{25} (h)**	2nd derivative (25,31)	10	0.95	0.77	0.84	0.82	3.7	0.00
**T_{30} (h)**	2nd derivative (9,19)	5	1.07	0.72	0.84	0.82	3.7	0.06
**T_{40} (h)**	2nd derivative (7,27)	4	1.25	0.64	0.99	0.77	3.4	0.08
**T_{50} (h)**	2nd derivative (5,19)	6	1.38	0.59	1.12	0.71	3.0	0.17

Ŧ 2nd derivative (w, s) refers to preprocessing with second derivative of the spectra using window/gap size of w, i.e., the number of data points across which the derivative is taken and segment, s, is the number of data points/segments across which smoothing/averaging is performed prior to derivative.
